# Impact of vaginal douching products on vaginal *Lactobacillus*, *Escherichia coli* and epithelial immune responses

**DOI:** 10.1038/s41598-021-02426-5

**Published:** 2021-11-29

**Authors:** Helai Hesham, Alissa J. Mitchell, Agnes Bergerat, Kristin Hung, Caroline M. Mitchell

**Affiliations:** 1 Division of Female Pelvic Medicine and Reconstructive Surgery, Massachusetts General Hospital, Boston, MA USA; 2 Vincent Center for Reproductive Biology, Massachusetts General Hospital, 55 Fruit St., Boston, MA 02114 USA; 3 Present Address: Division of Female Pelvic Medicine and Reconstructive Surgery, Columbia University Medical Center, New York, USA

**Keywords:** Bacterial infection, Urinary tract infection, Clinical microbiology

## Abstract

We compared the effect of commercial vaginal douching products on *Lactobacillus crispatus, L. jensenii, L. gasseri, L. iners*, *E. coli*, and immortalized vaginal epithelial cells (VK2). All studied douching products (vinegar, iodine and baking soda based) induced epithelial cell death, and all inhibited growth of *E. coli*. Co-culture of vaginal epithelial cells with any of the lactobacilli immediately following exposure to douching products resulted in a trend to less human cell death. However, co-culture of epithelial cells with *L. iners* was associated with higher production of IL6 and IL8, and lower IL1RA regardless of presence or type of douching solution. Co-culture with *L. crispatus* or *L. jensenii* decreased IL6 production in the absence of douches, but increased IL6 production after exposure to vinegar. Douching products may be associated with epithelial disruption and inflammation, and may reduce the anti-inflammatory effects of beneficial lactobacilli.

## Introduction

Between 10 and 40% of American women use vaginal douching products to treat vaginal discharge, odor or discomfort^1–4^. Intravaginal washing is associated with decreased vaginal colonization with beneficial lactobacilli^5^. Cross-sectional studies have demonstrated an association between douching and bacterial vaginosis (BV)^5,6^. Although women with recurrent vaginal infections are advised not to douche, studies of vaginal washing cessation have not demonstrated dramatic restoration of a *Lactobacillus*-dominant vaginal microbiota in women who stop the practice^7–9^. It is unclear whether douching causes the alteration in vaginal microbiota, is initiated as a response to a change in microbiota and symptoms or is simply a confounder associated with an unknown risk factor.

Urinary tract infections (UTI) are diagnosed in over 7 million women annually, and are most commonly caused by *E. coli*^10–12^. Vaginal *Lactobacillus spp.* are thought to be protective against *E. coli* due to bactericidal activity and there is an inverse association between the presence of vaginal lactobacilli and *E. coli* in women with recurrent UTI^13,14^. Women with BV (and thus, low levels of vaginal lactobacilli) are at an increased risk for UTI^15,16^. However, douching itself has not been well studied as a risk factor for UTI.

One way to assess the potential role of douching in altering the risk for UTI is to determine the impact of douching products on *E. coli* and commensal vaginal lactobacilli. A study examining the effect of vinegar-based douching products in vitro showed no inhibitory effects on either *L. gasseri or L. jensenii*, though it did demonstrate inhibition with iodine-based solutions^17^. However, there are no in vitro studies looking at the inhibitory effects of douching products on the most common vaginal *Lactobacillus* species (*L. crispatus* and *L. iners*), or *E. coli,* the most common pathogen isolated in UTI. In an effort to better understand how douching products impact the vaginal environment and potential risk for UTI, we co-cultured commercially available douches with the four most common vaginal *Lactobacillus spp.*, *E. coli* and vaginal epithelial cells and measured the effect of douches on bacterial growth and human cell viability and cytokine production.

## Results

### Effect of douching products on bacterial growth

**Figure 1:**
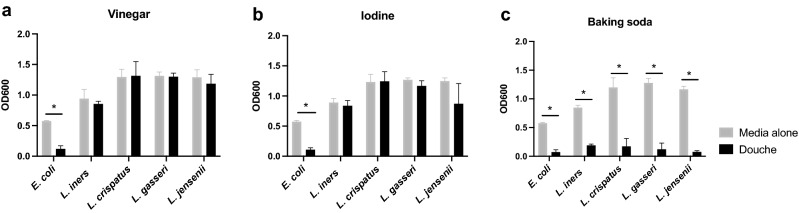
Impact of douching products on bacterial growth. Full strength vinegar (**a**), iodine (**b**) and baking soda-based (**c**) douching products were mixed 1:2 with broth culture of *E. coli* or one of four *Lactobacillus* species and growth compared by OD600 over 2 h (*E. coli*) or 24 h (lactobacilli). Results are presented as mean ± SD from at least two separate experiments.

We co-cultured *E. coli* and the four most common vaginal *Lactobacillus* species (*L. crispatus, L. iners, L. gasseri, L. jensenii*) with vinegar-, iodine-, and baking soda-based commercial douching products (Table 1). *E. coli* was grown aerobically in nutrient broth to mid-exponential phase, diluted to a standard starting OD600 of 0.1 and mixed with the different douching solutions or sterile saline. After 2 h at 37C, *E. coli* growth was inhibited by all products (*p* < 0.01) compared to the saline control (Fig. 1). *Lactobacillus* species were cultured anaerobically in MRS broth (*L. crispatus, L. gasseri, L. jensenii*) or MRS broth plus Isovitalex (*L. iners*), and co-incubated with douching solutions for 24 h at 37C. The only douching solution which inhibited growth of any *Lactobacillus* species was the baking soda-based product (Fig. 1). *E. coli* growth was inhibited by more dilute solutions of iodine and baking soda douching products, but *Lactobacillus* species were only inhibited by the undiluted product (Supplemental Fig. 1).

**Table 1:** Ingredients and pH of douching agents tested.

Product	Listed ingredients	pH
A	Water, Sodium Chloride, Vinegar, Sodium Benzoate	3.0
B	Citric acid, Edetate disodium, Water, Sodium benzoate, Sodiumlauryl sulfate, Trisodium phosphate, Povidone-Iodine (0.3%)	3.5
C	Sodium bicarbonate, Water	9.0

### Effect of douching products on vaginal epithelial cell viability and immune response

**Figure 2:**
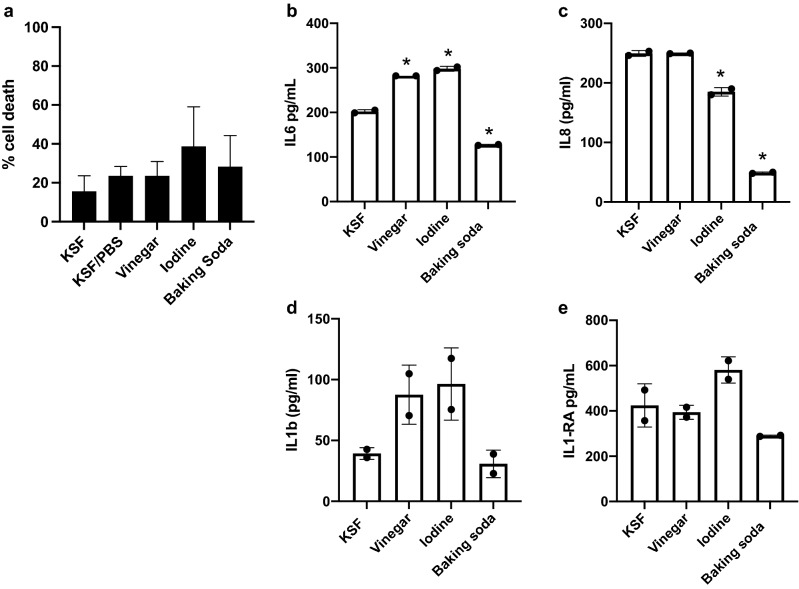
Impact of douching products on vaginal epithelial cells. An immortalized vaginal epithelial cell line was cultured with a 25% solution of douching product diluted in the cell culture medium for 1 h, followed by replacement with fresh media for 23 h. Cell death (**a**) was measured using an LDH assay and IL6 (**b**), IL8 (**c**), IL1β (**d**) and IL1-RA (**e**) were measured in supernatant by ELISA. Results are presented as mean ± SD from at least two separate experiments.

We then examined the effect of douching products on the vaginal epithelium by co-culturing immortalized vaginal epithelial cells (VK2) with douching solution diluted 1:4 in the cell culture media (keratinocyte serum-free (KSF) medium) for one hour. After the hour, media was aspirated and replaced with fresh culture medium and cultured for an additional 23 h. Human cell death, measured using a lactate dehydrogenase (LDH) assay, was higher after exposure to douching products, though this effect was not statistically significant (Fig. 2a). We evaluated cytokine expression in culture supernatant using ELISA. When compared to saline control, a one-hour exposure to iodine and baking soda suppressed production of IL8 (Fig. 2b). Both vinegar and iodine douching products induced greater production of IL6 and IL1β, while baking soda suppressed production (Fig. 2c,d). IL1RA was not significantly different between KSF alone and douching products (Fig. 2e).

### Effect of lactobacilli on epithelial cell viability and immune response

**Figure 3:**
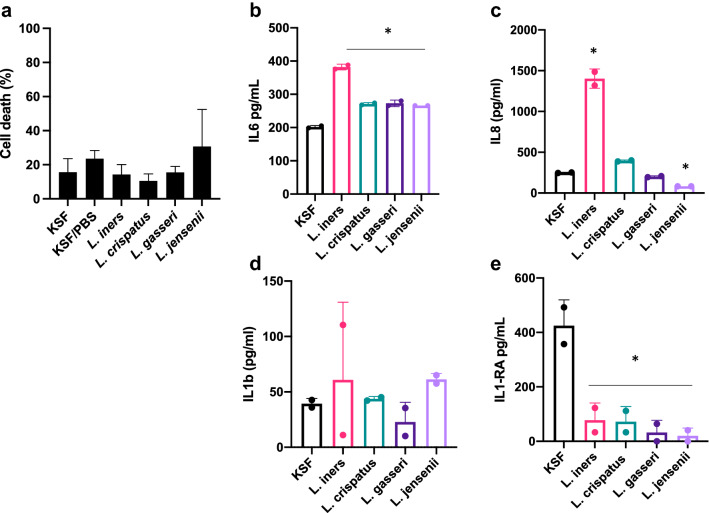
Impact of bacteria on vaginal epithelial cells. An immortalized vaginal epithelial cell line was cultured with a suspension of *Lactobacillus iners, L. crispatus, L. gasseri* or *L. jensenii* in cell culture medium (OD 0.1) for 24 h. Cell death (**a**) was measured using an LDH assay and IL6 (**b**), IL8 (**c**), IL1β (**d**) and IL1-RA (**e**) were measured in supernatant by ELISA. Results are presented as mean ± SD from at least two separate experiments.

Lactobacilli were resuspended in KSF at an OD of 0.1 and added to the human vaginal epithelial cell cultures for 24 h. There was no difference in cell death after exposure to any of the *Lactobacillus* spp. (Fig. 3a). When compared to KSF alone, co-culture with *L. iners* induced production of IL6 and IL8 (Fig. 3b,c) and suppressed production of IL1-RA (Fig. 3e). The other three species did not induce cytokine production (Fig. 3b–d), but all suppressed production of IL1-RA (Fig. 3e).

### Impact of lactobacilli on epithelial cell response to douching products

**Figure 4:**
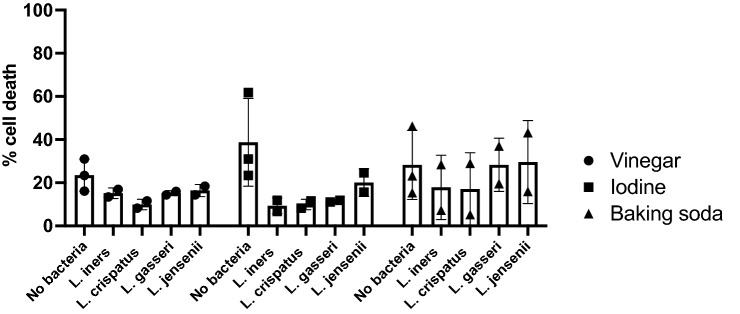
Impact of bacteria on vaginal epithelial cell death with douching exposure. An immortalized vaginal epithelial cell line was cultured with a 25% solution of douching product diluted in the cell culture medium for 1 h, followed by replacement with a suspension of *L. crispatus* or *L. iners* (OD 0.1) in cell culture media for 23 h. Cell death was measured with an LDH assay and values compared by ANOVA. Results are presented as mean ± SD from at least two separate experiments.

We then examined how exposure to a douching solution impacted the epithelial cell response to commensal lactobacilli. Co-culture with either *L. crispatus* or *L. iners* for 23 h after a 1-h exposure to douching solutions resulted in a trend to less human cell death compared to cell culture media alone, though this did not reach statistical significance (Fig. 4).

**Figure 5:**
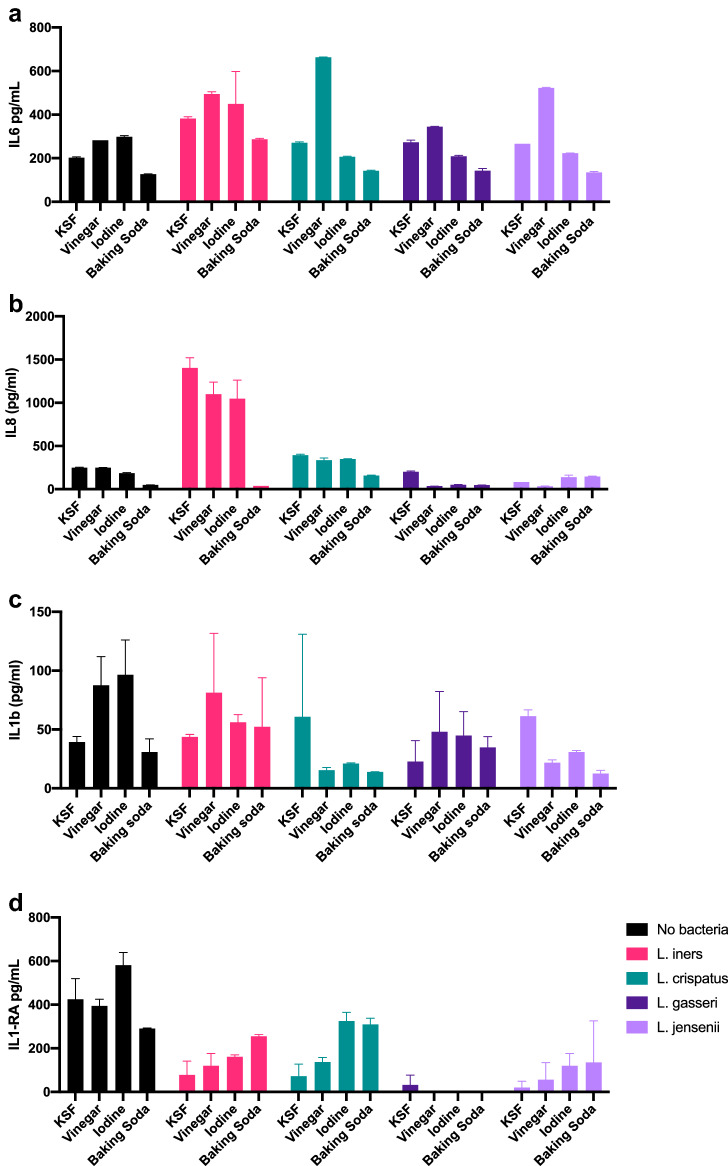
Impact of bacteria on vaginal epithelial cell death immune response to douching exposure. An immortalized vaginal epithelial cell line was cultured with a 25% solution of douching product diluted in the cell culture medium for 1 h, followed by replacement with a suspension of *L. iners, L. crispatus, L. gasseri or L. jensenii* (OD 0.1) in cell culture media for 23 h. Supernatant was used to measure IL6 (**a**), IL8 (**b**), IL1β (**c**) and IL1-RA (**d**) with ELISA, and values compared by ANOVA. Results are presented as mean ± SD from at least two separate experiments.

Whether bacteria were present or not, baking soda douche suppressed production of IL6 and IL8 compared to media alone (Fig. 5a,b). *L. crispatus* suppressed production of IL1b across conditions, though this did not reach statistical significance (Fig. 5c). When baking soda douche was followed by *L. iners* or *L. crispatus*, cells produced higher levels of IL1RA (Fig. 5d). Pre-incubation with vinegar induced higher levels of IL6, most significantly with subsequent exposure to *L. crispatus*. Pre-treatment with douching products suppressed production of IL8 after exposure to *L. iners*, though overall values were still higher than after exposure to other lactobacilli.

## Discussion

Between 9 and 48% of women in the US report use of vaginal douching products, despite recommendations from health care providers against douching^1,3^. Washing practices have been associated with increased risk for BV, persistent human papillomavirus (HPV) infection, and decreased vaginal colonization with hydrogen peroxide-producing *Lactobacillus*^5,6,18^. In our in vitro model, all three types of commercial douching solutions tested inhibited growth of the common uropathogen *E. coli,* while vinegar- and iodine-based douches did not inhibit commensal lactobacilli. All douches were toxic to vaginal epithelial cells, but co-culture with commensal *Lactobacillus spp.* decreased that effect. Our study is one of the first to examine the common species, *L. iners*, which does not grow in the traditional *Lactobacillus*-selective medium MRS. We found that *L. iners* is more inflammatory than other species, which is in keeping with the finding that *L. iners*-dominant communities have more inflammation in vivo than those dominated by *L. crispatus*^19,20^. However, all *Lactobacillus* species mitigated the toxicity of douching products on epithelial cells.

While many cross-sectional studies show an association between bacterial vaginosis and douching^4,21,22^, some do not^23,24^, which has raised questions about the causal association between douching and BV. Cross-sectional studies demonstrate lower detection of vaginal lactobacilli in women who douche^25^. A small longitudinal study of a douching cessation intervention demonstrated an increase in vaginal lactobacilli after cessation, though this finding was not statistically significant^7^. Douching has been associated with increased risk for adverse reproductive health outcomes such as pelvic inflammatory disease and endometritis^26^, or HIV acquisition^27^. Recent studies demonstrate higher numbers of HPV types or higher prevalence of high risk subtypes in women who douched in the past 6 months^18,28^. Few studies have examined the contribution of douching to risk for UTI. In a case control study of young Seattle women with and without recurrent UTI, douching was reported by only 11% of participants and was not different between cases and controls^29^.

Few studies have examined the effect of vaginal douching solutions on uropathogens and commensal vaginal bacteria and none have tested baking soda-based douching solutions. A previous in vitro study demonstrated that vinegar based douching solutions inhibited multiple vaginal pathogens but had no impact on vaginal lactobacilli. However, the same study showed that iodine-based douching products inhibited *L. gasseri* but not *L. jensenii*, which contrasts with our findings^17^. A separate evaluation of a vinegar-based douche also demonstrated no impact on growth of *L. crispatus *in vitro^30^. An in vivo comparison of vinegar vs. iodine-based douching solutions, used daily for 7 days, demonstrated a decrease in detection of *Lactobacillus* species by culture after iodine-based douche but not vinegar-based. *E. coli* was infrequently detected in all participants, regardless of douching^31^. The acidity of a vinegar-based douche could be hypothesized to be beneficial for the vaginal environment, mimicking the low pH associated with a *Lactobacillus*-dominant bacterial community. However, a study of a lactic acid based vaginal douching product demonstrated higher prevalence of a diverse vaginal microbial community after use, which is the opposite of what one might expect^32^. In vitro, lactic acid-based vaginal douches, which also included several other ingredients, inhibited clinical *Lactobacillus* strains^33^. As commensal lactobacilli have shown to have bactericidal activity against common uropathogens such as *E. coli*, loss of lactobacilli due to douching could increase UTI risk, though this might be balanced by the negative effects of many douching products on *E. coli* growth^34,35^.

Our study, and others, demonstrate in vitro that vaginal douching products increase vaginal epithelial cell death and secretion of pro-inflammatory cytokines, suggesting the potential for epithelial disruption^30^. Additionally, and of concern, is our finding that after exposure to vinegar-based douche, *L. crispatus* and *L. jensenii*—two classic beneficial lactobacilli—induce greater production of the pro-inflammatory cytokine IL6. This result may be due to less cell death, and so greater ability to produce cytokines, but if so we would expect to see a similar pattern with *L. iners* and *L. gasseri*. In vivo, analysis of vaginal fluid cytokine levels demonstrates higher levels of pro-inflammatory cytokines in women who use douching products. However, the type of vaginal douching solution was not specified^36^.

Other common vaginal personal care products, such as lubricants, have also been shown to limit growth of both uropathogens and commensal lactobacilli^34,37^. Unlike douches, many of those products contain antimicrobial preservatives such as chlorhexidine or parabens, or detergents such as nonoxynol-9. Lubricant products have also been shown to impact epithelial barrier integrity, and reduce adherence of lactobacilli to vaginal epithelial cells^37–39^. The douching products we tested also include other compounds, which may contribute to the effects seen; citric acid (included in the iodine-based douche) has been associated with epithelial toxicity, as has benzoic acid (included in both iodine- and vinegar-based douches) to a lesser extent^40^.

Limitations of this study include the lack of a robust model for human vaginal microbial colonization. The vaginal epithelium is composed of layers of cells with basal, parabasal, squamous flat cells and intermediate cells. Disruption of the epithelium could lead to exposure of stroma or antigen-presenting cells which might have different immune responses. Our co-culture model does not mimic this multilayer system. Also, our co-culture model does not include the mucous normally found in the in vivo setting, which could serve as a protective barrier to the effects of douching products—for both epithelial cells and bacteria. However, there are no animal models of the vaginal ecosystem that include *Lactobacillus*-dominant microbiota in which to conduct these studies^41^.

Our study highlights the potential negative impacts of douching solutions through induction of epithelial cell death and inflammation. Douches at either end of the pH spectrum (i.e. baking soda and vinegar) had different but consistently negative effects on either human cells or commensal lactobacilli. Our results suggest possible mechanisms through which vaginal douching could potentially increase acquisition of all genitourinary infections, including UTI, and support clinical recommendations to avoid douching.

## Materials and methods

### Douche/bacteria co-culture

Three vaginal douching products with different active ingredients were purchased online or at local drug stores within the United States. The ingredients of the douching solutions as well as their pH values are listed in Table 1. Five species of bacteria were grown in broth to mid-exponential phase: *E. coli* (clinical strain, obtained from the clinical lab at MGH, reported as from a woman with acute cystitis), *Lactobacillus crispatus* (obtained through BEI Resources, NIAID, NIH as part of the Human Microbiome Project: *Lactobacillus crispatus*, Strain JV-V01, HM-103), *L. jensenii* (ATCC 25258), *L. gasseri* (ATCC 33323), *L. iners* (ATCC 55195). *E. coli* was grown in nutrient broth to mid-exponential phase, diluted to a standard starting OD600 and mixed with the different douching solutions or sterile saline. *E. coli* experiments were conducted aerobically, at 37C with gentle agitation. *Lactobacillus* species were cultured in MRS broth (*L. crispatus, L. gasseri, L. jensenii*) or MRS broth plus Isovitalex (*L. iners*)^42^. Co-culture experiments were performed by mixing 400 uL of the bacterial broth cultures and 200 uL of douches in varying concentrations (100%, 75%, 50%, and 25%, diluted with saline) in a 48 well plate. *Lactobacillus* experiments were conducted in an anaerobic chamber, at 37C, and the plates were kept still. Each combination was tested in triplicate and compared to a saline control. Change in optical density (OD600) was measured over 2 h for *E. coli* and 24 h for Lactobacilli to assess growth using a Gen 5 2.09 Biotek microplate reader.

### Vaginal epithelial cell/douche/bacteria co-culture

Vaginal epithelial cells (VK2 E6/E7, ATCC) were seeded at 40,000 cells/well in a 96-well plate, in Keratinocyte-Serum Free medium (KSF) (with BPE and EGF, but no antibiotics), and allowed to grow overnight to confluence. Vaginal cells were then exposed to dilute (25%) douching product or fresh media for 1 h. This concentration and duration of exposure was chosen because of prior data showing that exposure to undiluted douching products inhibited vaginal epithelial cell viability^30^, and our own data showing significant human cell death at higher concentrations or for longer duration (Supplemental Fig. 2). Supernatant was aspirated, and vaginal cells were then exposed to a fresh KSF or a suspension of one of the four commensal *Lactobacillus* species in KSF for 23 h (at OD 0.1). At the end of the exposure, supernatant was aspirated and used to measure LDH or frozen until used for ELISA. Vaginal epithelial cell death was quantified using a lactate dehydrogenase assay (Invitrogen, Waltham, MA). Pro-inflammatory cytokine levels (IL6, IL8, IL1β, IL1RA) were measured using ELISA (R&D Systems, Minneapolis, MN).

### Statistical analysis

Change in OD600 between time 0 and the end of the bacterial/douche co-culture was compared across concentrations and media control for each product using ANOVA. Cell death was compared between individual douching product or *Lactobacillus* species and KSF control by student’s T-test. Cytokine values were compared between conditions using one-way or two-way ANOVA followed by Tukey’s multiple comparison test, as appropriate.

## Supplementary Information

Supplementary Figure 1.

Supplementary Figure 2.
