# Supplementary material for: Impact of vaginal douching products on vaginal Lactobacillus, Escherichia coli and epithelial immune responses
Source: Sci Rep. 2021 Nov 29;11:23069. doi: 10.1038/s41598-021-02426-5 (PMC8629978; doi:10.1038/s41598-021-02426-5)
Supplement: Supplementary file 1 — Supplementary Figure 1. [file 41598_2021_2426_MOESM1_ESM.pdf]

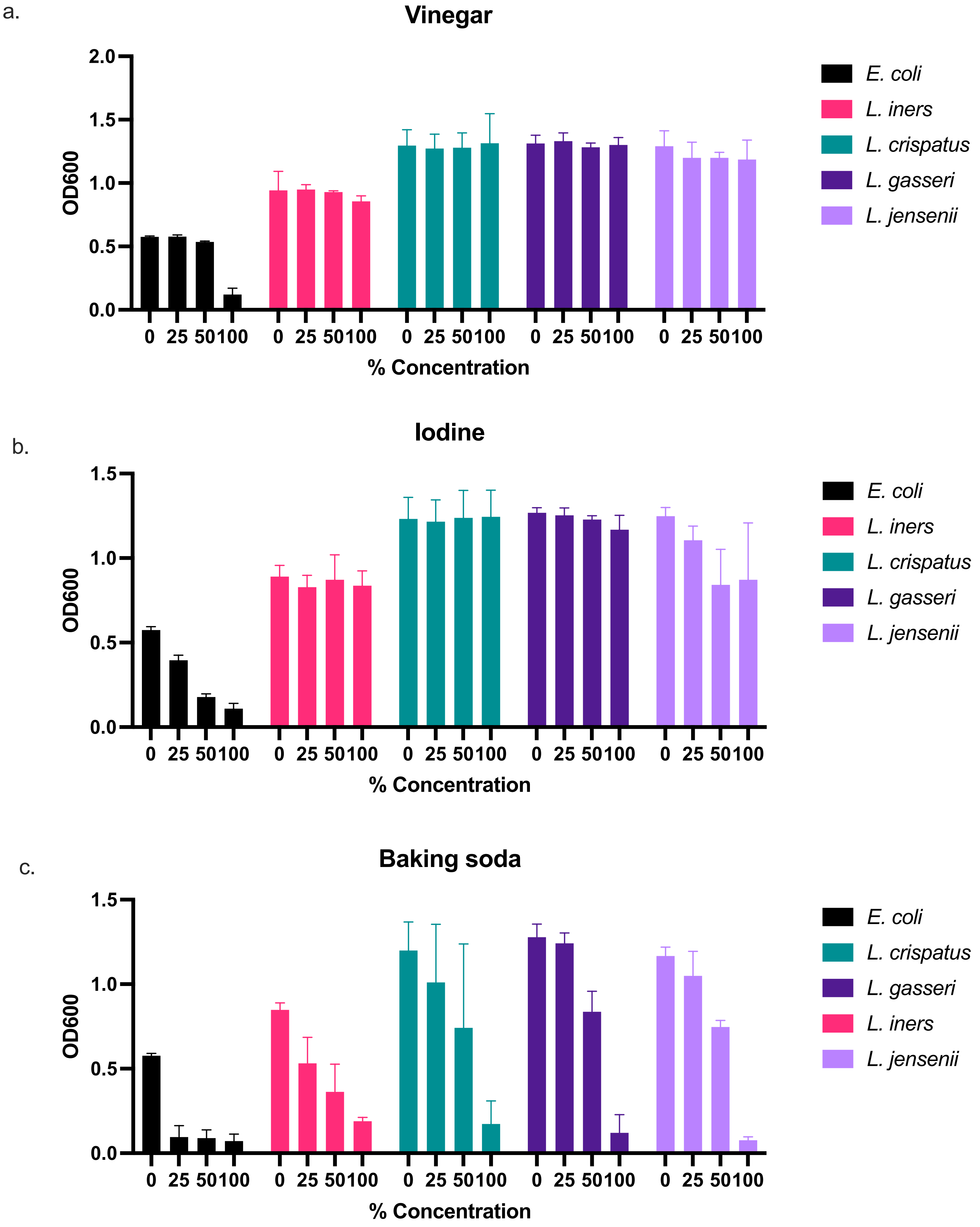

Supplemental Figure 1: Impact of douching products on bacterial growth. Solutions of vinegar- (a), iodine- (b) and baking soda-based (c) douching products at 25%, 50% and 100% were mixed 1:2 with broth culture of *E. coli* or one of four *Lactobacillus* species and growth compared by OD600 over 2 hours (*E. coli*) or 24 hours (lactobacilli).
