# Supplementary material for: Impact of vaginal douching products on vaginal Lactobacillus, Escherichia coli and epithelial immune responses
Source: Sci Rep. 2021 Nov 29;11:23069. doi: 10.1038/s41598-021-02426-5 (PMC8629978; doi:10.1038/s41598-021-02426-5)
Supplement: Supplementary file 2 — Supplementary Figure 2. [file 41598_2021_2426_MOESM2_ESM.pdf]

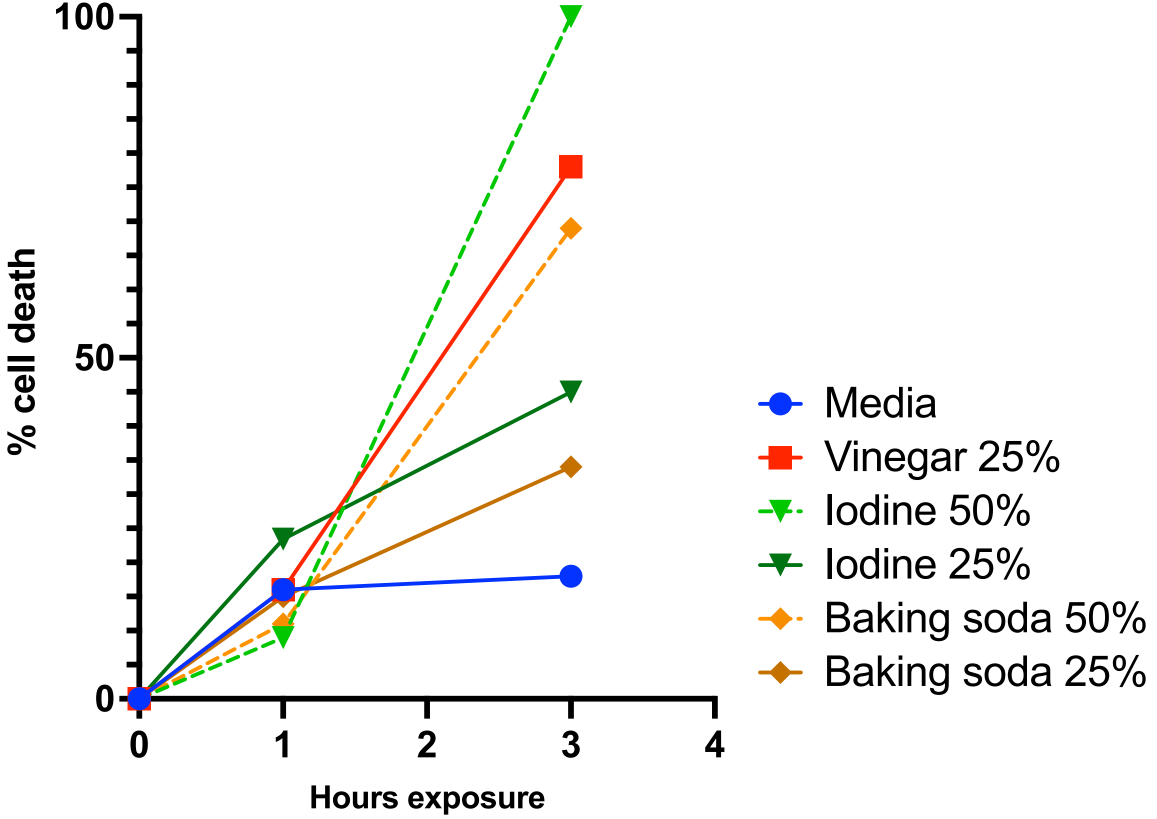

Supplemental Figure 2: Impact of douching products on human vaginal epithelial cell viability. Solutions of 25% vinegar, and 25% or 50% baking soda or iodine douching products were incubated with human vaginal epithelial cells for 1 or 3 hours, and then cell death measured using an LDH assay.
